# Cell adhesion molecules and immunotherapy in advanced non-small cell lung cancer: Current process and potential application

**DOI:** 10.3389/fonc.2023.1107631

**Published:** 2023-02-21

**Authors:** Hongjian Yang, Yuxi Miao, Zhaojin Yu, Minjie Wei, Xue Jiao

**Affiliations:** ^1^ Innovative Institute, China Medical University, Shenyang, China; ^2^ Department of Pharmacology, School of Pharmacy, China Medical University, Shenyang, China; ^3^ Liaoning Key Laboratory of Molecular Targeted Anti-Tumor Drug Development and Evaluation, Shenyang, China; ^4^ Liaoning Cancer Immune Peptide Drug Engineering Technology Research Centre, Shenyang, China; ^5^ Shenyang Kangwei Medical Laboratory Analysis Co. LTD, Shenyang, China

**Keywords:** non-small cell lung cancer, monoclonal antibody, immunotherapy, immune checkpoint inhibitor, cell adhesion molecules

## Abstract

Advanced non-small cell lung cancer (NSCLC) is a severe disease and still has high mortality rate after conventional treatment (e.g., surgical resection, chemotherapy, radiotherapy and targeted therapy). In NSCLC patients, cancer cells can induce immunosuppression, growth and metastasis by modulating cell adhesion molecules of both cancer cells and immune cells. Therefore, immunotherapy is increasingly concerned due to its promising anti-tumor effect and broader indication, which targets cell adhesion molecules to reverse the process. Among these therapies, immune checkpoint inhibitors (mainly anti-PD-(L)1 and anti-CTLA-4) are most successful and have been adapted as first or second line therapy in advanced NSCLC. However, drug resistance and immune-related adverse reactions restrict its further application. Further understanding of mechanism, adequate biomarkers and novel therapies are necessary to improve therapeutic effect and alleviate adverse effect.

## Introduction

Nowadays, cancer is the second leading cause of death, the first leading cause of disability-adjusted life years (DALYs) loss and will probably be the first leading cause of death in 2060 (1). According to WHO GLOBOCAN 2020 database, lung cancer is the second most common cancer after breast cancer and causes more dead cases than any other cancer. To make matters worse, smoking and air pollution are still increasing in many developing countries (2). The affordable and accessible cigarettes and chronic health effect of nicotine leads to long-term dependence of smoking and an elevation of lung cancer risk (3). Lung cancer is a kind of highly heterogenous disease and can be categorized into two main groups: NSCLC (85%) and SCLC (15%) (4). In this review, we will mainly talk about advanced NSCLC which is unresectable for most patients. Chemotherapy and radiotherapy also provide modest efficacy (5). Target therapy has been invented and adapted as first-line treatment for certain genotype NSCLC patients, which can inhibit tumor progression and significantly improve prognosis by blocking abnormal signaling pathway (6). However, targeted therapy is not effective forever. For example, NSCLC will develop resistance to EGFR tyrosine kinase inhibitors (TKIs) after a median of 10 to 14 months treatment and effective treatment has not been defined except Osimertinib for the T790M mutation (7, 8).

Studies have showed that tumor immune evasion participates in the whole course of NSCLC rather than only in advanced stage. Immune cells will be activated at early stage with an elevation of anti-tumor cells (e.g., NK cells and CD8 ^+^ cells) but soon inhibited at advanced stage (e.g., the transitory activation of CD4 ^+^ and CD8 ^+^ T cells, from Th1 to Th2, the increase of regulatory T cells (Tregs)) (9, 10). Cell adhesion molecules mediate the contact and binding between cells or between cells and extracellular matrix. They can be divided into five groups: integrins, selectins, cadherins, immunoglobulin superfamily and mucin-like vascular addressin and are crucial for the formation of tumor microenvironment (11). The overexpression of CTLA-4, PD-1, and PD-L1 can negatively regulate anti-tumor immunity (9). Low expression of E-cadherin is associated with poor prognosis and LFA-1 participates in T cell activation and migration (12, 13). In this review, we will mainly talk about how cell adhesion molecules participate in tumor progression and therapies for cell adhesion molecules.

Immune checkpoint inhibitors (ICIs) can blockade negative costimulatory molecules and reverse the tumor-induced immunosuppression. ICI is the only immunotherapy that showed clinical benefit in NSCLC and has showed better long-term survival compared with chemotherapy and radiotherapy (14, 15) (**Table 1**). Despite of these advantages, ICIs also have its limitations. Primary and secondary resistance to ICIs is common and around 40–50% of lung cancer patients experienced disease deterioration during the first cycles of immunotherapy (16). Various immune-related adverse effects (irAEs) decrease life quality and even leads to treatment failure or death (17). Combined therapy, biomarkers and novel ICIs are promising to increase the response rate and treatment efficacy (18). After adequate treatment, retreatment is possible and may be beneficial for irAE patients who had no treatment response before irAEs onset (19). Cell adhesion molecules are also potential targets for other immunotherapies (e.g., tumor vaccines, CAR-T) which may be helpful to enhance immunotherapy efficacy (20, 21).

**Table 1:** Overview of anti-PD-1/PD-L1 and CTLA-4 monoclonal antibodies approved by the FDA with NSCLC indication as of November 2022.

Drug	Trademark	Description	Target	Biologic License Application (BLA)	Manufacturer	Approved by the FDA (MM/DD/YYYY)	Original indications
Pembrolizumab	Keytruda	Humanized IgG4	PD-1	125514	Merck Sharp & Dohme	09/04/2014	Unresectable or metastatic melanoma Melanoma with involvement of lymph node(s) following complete resection
Nivolumab	Opdivo	Human IgG4	PD-1	125527	Bristol-MyersSquibb	03/04/2015	Advanced squamous NSCLC
Atezolizumab	Tecentriq	Humanized IgG1	PD-L1	761034	Genentech Inc.	05/18/2016	Locally advanced or metastatic urothelial carcinoma
Durvalumab	Imfinzi	Human IgG1	PD-L1	761069	AstraZeneca UK Ltd.	05/01/2017	Advanced or metastatic urothelial carcinoma
Cemiplimab	Libtayo	Recombinant human IgG4	PD-1	761097	Regeneron Pharmaceuticals	09/28/2018	Metastatic cutaneous squamous cell carcinoma or patients with locally advanced cutaneous squamous cell carcinoma who are not candidates for surgery
Dostarlimab	Jmeperli	Humanized IgG4	PD-1	761174	GlaxoSmithKline LLC	04/22/2021	Recurrent or advanced endometrial cancer patients with mismatch repair deficient (dMMR) has progressed on or following prior treatment with a platinum-containing regimen
Tremelimumab	IMJUDO	Human IgG2	CTLA-4	761289	AstraZeneca Pharmaceuticals LP	10/21/2022	Unresectable hepatocellular carcinoma
Ipilimumab	Yervoy	Human IgG1	CTLA-4	125377	Bristol-Myers Squibb	03/25/2011	Advanced melanoma

## Effects of cell adhesion molecules on the progression of NSCLC

Cell adhesion molecules plays a crucial role in tumor progression. MUC-1 expression on tumor cells promotes metastasis by binding to intercellular adhesion molecule-1 (ICAM-1) in endothelial cells. Integrins increases the number of tumor-associated macrophages (TAMs), and both integrin and TAMs are associated with epithelial-to-mesenchymal transition of epithelial tumor cells, which leads to loss of epithelial markers and increase of mesenchymal markers. As a result, tumor cells are more easily to metastasis (22, 23). Some cell adhesion molecules also mediate tumor immune evasion, such as cytolytic T lymphocyte-associated protein-4 (CTLA-4) and programmed cell death protein-1 (PD-1)/programmed cell death-ligand 1 (PD-L1), which are also called immune checkpoint (24). High expression of certain cell adhesion molecules in Tregs may lead to Tregs infiltration and immunosuppression at tumor site (11). For NSCLC, higher immune score was associated with better prognosis in adenocarcinoma, while this relation was not found in squamous cell carcinoma (25). In this review, we will mainly talk about aberrant cell adhesion molecules in NSCLC.

## LFA-1 and ICAM-1

LFA-1 (CD11a/CD18) belongs to integrin family and mainly expressed by blood cells and ICAM-1 is an important ligand for LFA-1. Besides the adhesive function like other integrins, LFA-1 has “outside-in” and “inside-out” bidirectional signaling pathways, it also has different conformations, from folded low-affinity to extended high-affinity. LFA-1 has complex effect in tumor progression. On the one hand, it promotes the differentiation of T cells, mediates cytotoxic anti-tumor response and is necessary for lymphocyte infiltration; on the other hand, leukocytes infiltration may promote tumor progression, such as Tregs (26). Like LFA-1, ICAM-1 is also important in tumor progression. ICAM family belongs to immunoglobulin superfamily and participates in immune responses and intracellular signaling. Currently, there are five known ICAMs (ICAM-1 to ICAM-5) (27).

LFA-1 is a key T cell integrin and participates in regulation of T cell activation and migration (12). LFA-1 rather than CD28 enhanced the impact of TCR clustering, which is consistent with the localization effect of LFA-1 (28, 29). This process is largely dependent on the activation of multiple kinases and adaptor proteins, such as phosphoinositide 3-kinase, Crk protein and ERK pathways (30, 31). The binding of LFA-1 and ICAM-1 participates in T cell differentiation into specific phenotype (32). The binding of LFA-1 with high density ICAM-1 can directly facilitate cytokine secretion by increasing cytoplasmic Ca^2+^ and ERK phosphorylation in invariant natural killer T cells (33). LFA-1 is also necessary for extracellular ISG15 to mediate IL-10 and IFN-γ secretion (34). PI3Kδ also facilitates T cell activation by increasing LFA-1/ICAM-1 interaction (35). And G-CSF inhibits LFA-1/ICAM-1-mediated CD4 ^+^ T cells inflammatory cytokines secretion by down-regulating Lck and ZAP-70 (36). CD8 ^+^ T cell has intracellular LFA-1 storage, which can be transported to cell surface after antigenic stimulation and is an important mechanism for LFA-1 to regulate T cell differentiation (37). Although ICAMs is not necessary for Th1 differentiation, LFA-1/ICAM-1 can promotes Th1 polarization through Notch pathway (38, 39). LFA-1 also plays an important role in normal T follicular helper cells function through upregulation of Bcl-6 and DOCK8 (40, 41).

In tumor progression, aberrant ICAM-1 expression and impaired LFA-1/ICAM-1 function has been observed. Increased sICAM-1 concentration in the exhaled breath condensate has been observed in NSCLC patients compared with that of healthy people and COPD patients and sICAM-1 concentration was significantly decreased after resection (63.4± 26.0 ng/mL vs 44.0 ± 17.7 ng/mL, p < 0.01) (42). A meta-analysis revealed that higher concentration of circulating soluble ICAM-1 (sICAM-1) was associated with more advanced disease stage and poorer prognosis in NSCLC. This may be explained by the impaired ICAM-1/LFA-1 interaction due to the binding of sICAM-1 and LFA-1, tissue damage and angiogenesis caused by increased sICAM-1 (43). Galectin can disrupt the formation of functional tumor-infiltrating T cells synapse by preventing LFA-1 recruitment and the affinity regulation (44). ICAM-3 promoted tumor metastasis by binding to LFA-1 in NSCLC cells (45). LFA-1/ICAM-1 also disrupts CD8 ^+^ T cells recirculation by promoting tumor tissues aggregation (46). NSCLC cells can adhere to vertebral bone marrow endothelial cells through CX3CL1/ICAM-1/LFA-1 pathway and lead to spinal metastasis (47). On the other hand, LFA-1/ICAM-1 interaction has potential anti-tumor effect because it is an alternative costimulatory signaling to activate anti-tumor cytotoxic T cells (48). NSCLC cells exhibited an IFN-γ-dependent ICAM-1 upregulation activated by EGFR CAR-T cells. This ICAM-1 upregulation permitted EGFR CAR-T cells to move from tumor edge to center, and blockade of ICAM-1/LFA-1 disrupted the CAR-T cell tumor infiltration (49). Impaired ICAM-1 upregulation on alveolar macrophages in patients with NSCLC after IFN-γ stimulation had also been observed, which might disrupt the normal function of alveolar macrophages and more studies are needed due to the small sample size and unclear mechanisms of this study (50). This contradiction has also been observed in other studies. Cannabinoids was reported to inhibit lung cancer cell invasion and metastasis by upregulating ICAM-1 *in vitro* (51). However, studies found that low level of ICAM-1 is associated with longer progression-free survival (PFS) and overall survival (OS). This may be contributed to the relation between aggressive NSCLC histological subtypes and high ICAM-1 expression. (e.g., adenocarcinomas and undifferentiated carcinomas) (52, 53).

## CTLA-4

CTLA-4 (CD152) is a kind of surface molecule in T cells and associated with immune regulation. Both CTLA-4 and CD28 can bind to B7-1 and B7-2. CD28 participates in the co-stimulation of T cells in the interaction between antigen presenting cells and T cells, while CTLA-4 has a higher affinity than CD28 and inhibits the activation of T cells. Therefore, CTLA-4 is highly expressed in activated T cells and Tregs in order to avoid autoimmune disease. However, CTLA-4 is activated in NSCLC and leads to immunosuppression and tumor immune evasion. These indicate that the blockage of CTLA-4 may be a promising immunotherapy target (25).

Ipilimumab is a CTLA-4-targeted monoclonal antibody and had been approved by food and drug administration (FDA) in metastatic melanoma. However, ipilimumab showed less effect compared with anti-PD-1 therapy when combined with radiotherapy in metastatic NSCLS patients (54). A meta-analysis showed that the combination of ipilimumab and chemotherapy didn’t improve OS compared to pure chemotherapy and were associated with more immune-related toxicities (55). Therefore, anti-CTLA-4 therapy is greatly restricted and has not been approved for NSCLC treatment (56).

## PD-1/PD-L1

**Figure 1:**
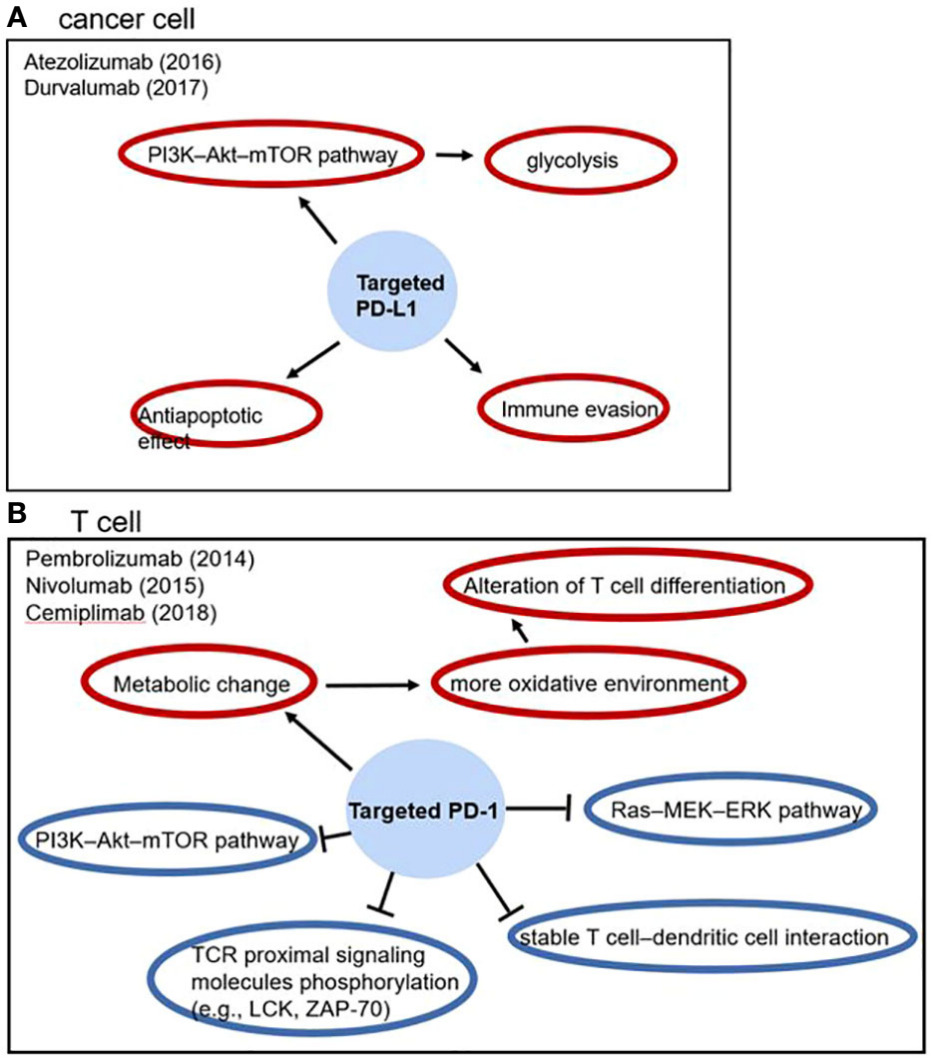
The effect of PD-1/PD-L1 in tumor progression and anti-PD-1/PD-L1 drugs, blue circles represent inhibitory effects and red circles represent stimulatory effects.

PD-1/PD-L1 is an immune checkpoint which is related to inhibitory immune regulation. PD-1 is expressed in a variety of immune cells especially T cells and has two ligands: PD-L1 and PD-L2. In cancer, overexpression of PD-L1 contributes to T cells hypofunction and apoptosis (57). For T cells, PD-1/PD-L1 ligation inhibits TCR proximal signaling molecules phosphorylation, PI3K–Akt–mTOR pathway and Ras–MEK–ERK pathway, it also contributes to dysfunction of T cell–dendritic cell interaction and metabolic alteration; for cancer cells, PD-L1 leads to antiapoptotic effect, immune evasion, PI3K–Akt–mTOR pathway activation and glycolysis, which facilitate cancer cell survival (**Figure 1**) (58). Therefore, PD-1/PD-L1 inhibitor blocks this process and shows satisfactory prognosis improvement in NSCLC (14).

PD-1/PD-L1 is a distinct target for NSCLC and has little cross-resistance with other kinds of anti-tumor agents. Clinical benefit of Osimertinib (a third generation EGFR TKI) is independent of PD-L1 expression level (59). PD-1 inhibitors are effective for NSCLCs with high PD-L1 expression regardless of EGFR genotype (60). PD-L1 expression is also failed to be a prognostic factor for advanced NSCLC patients with chemotherapy (61). 

## E-cadherin

The cadherin family is a class of cell adhesion molecules that mediates intercellular adhesion (62). Cadherin, especially E-cadherin, plays an important role in contact inhibition through Hippo, Wnt, TGF-β, NF-κB and other signal pathways. And downregulation of E-cadherin expression is associated with tumor progression and metastasis (63). Tissue resident memory T cell is a kind of tumor-specific T cells which can binds to E-cadherin and mediates T-cell receptor-dependent cytotoxic effect in NSCLC (64). The upregulation of E-cadherin and downregulation of N-cadherin induced by BRAF-activated non-coding RNA overexpression is also associated with epithelial to mesenchymal transition inhibition in lung cancer (65). And E-cadherin gene promoter hypermethylation is related to an elevated risk of NSCLC (66).

The expression level of E-cadherin and other relevant molecules may be promising biomarkers for NSCLC prognosis. Low expression of E-cadherin is closely associated with poor prognosis and biological behavior such as advanced clinical stage and lymph node metastasis, while high expression of paired related homeobox 1 (PRRX1) and low zinc expression of finger E-box binding homeobox 1 (ZEB1) are related to high E-cadherin expression and low level of epithelial-mesenchymal transition and tumor angiogenesis in NSCLC patients. These findings suggest that PRRX1 and ZEB1 may also be potential prognostic biomarkers and therapeutic targets for NSCLC (13, 67). The modification of E-cadherin can also affect the tumor progression. FUT8 gene encodes α-1,6-fucosyltransferase, which is the only enzyme for core-fucosylation on N-glycoproteins. Upregulation of FUT8 leads to an increase of core fucosylated E-cadherin and inhibits normal E-cadherin function. As a result, cancer metastasis is promoted. This is consistent with the correlation that upregulation of FUT8 is associated with increased tumor metastasis and worse survival in NSCLC, indicating that FUT8 may also be a potential prognostic biomarker for NSCLC (68).

## MUC-1

Mucin 1 (MUC-1) is an overexpressed glycoprotein and associated with cancer cell proliferation, migration and invasion in NSCLC. MUC1 consists of an extracellular N-terminal subunit (MUC1-N) and a transmembrane MUC1 C-terminal subunit (MUC1-C). From precancerous lesions to invasive carcinoma, upregulation of MUC-1 expression has been observed in patients with NSCLC (69). Studies showed that MUC1 was regulated by STAT3 and MUC1-C promoted the self-renewal of NSCLC cells *via* LIN28B/let-7/HMGA2 axis (70, 71). MUC1-C enhanced MYC expression in KRAS mutant NSCLC cells *via* WNT/β-catenin (CTNNB1) pathway activation, forming MUC1-C/β-catenin/TCF4 complexes on the MYC promoter and recruiting the p300 histone acetylase (EP300) to enhance histone H3 acetylation and MYC gene transcription. And MUC1-C inhibition contributed to an inhibition of MYC gene expression, tumor cell survival and tumor growth in KRAS mutant NSCLC cells (72). MUC1-C suppression was associated with inhibition of epithelial-mesenchymal transition (EMT) and KRAS independence in KRAS mutant NSCLC cells, which contributed to tumor growth inhibition (73). MUC1-C blockade also contributed to EGFR (T790M), AKT and ERK signaling suppression and survival inhibition in NSCLC cells with mutant EGFR (74). Silencing of MUC1 also enhanced the anti-tumor efficacy of paclitaxel against paclitaxel-resistant cell line A549/PR in NSCLC *via* Bax and Caspase-3 upregulation and Bcl-2 downregulation (75). MUC1 is also associated with tumor associated macrophage-induced lung cancer stem cell progression (76). *In vitro*, downregulation of MUC-1 was associated with AKT and ERK suppression, decreased VEGF and VEGF-C, tumor cell proliferation inhibition and increased cell apoptosis in NSCLC (77). Upregulation of MUC-1 after EGFR-targeted therapy has been observed, which is associated with PI3K/AKT/mTOR and JAK2/STAT3 pathways. This finding indicates that serum MUC-1 may be a novel biomarker for anti-EGFR therapy monitoring (78).

## Other molecules

CD44 is a non-kinase transmembrane glycoprotein and associated with tumor progression, metastasis and drug resistance in NSCLC (79, 80). High CD44v expression in tumor was related to shorter overall survival (p<0.001) and recurrence-free survival (p<0.001) after curative resection for patients with NSCLC (80). CD44s expression was related to lymph node metastases (p=0.007) and CD44v6 expression was more related to tumor size (p=0.0032) in lung adenocarcinoma (81). A study demonstrated that CD44 enhanced PD-L1 expression partly by the cleaved intracytoplasmic domain of CD44 bound to the consensus CD44-ICD binding site on the regulatory region of the PD-L1 locus in NSCLC (82). CD44 is also a downstream molecule upregulated by Notch3 in enhancing stem-like property in NSCLC cells (83). A study showed that CD44 facilitated CD133+CD44+ lung cancer stem cells metastasis *via* Wnt/β-catenin-FoxM1-Twist signaling (84). *In vitro*, overexpression of CD44 promoted NSCLC cell proliferation and colony formation and vice versa (85). Serglycin induces NSCLC cell migration by binding to the GAG motif to CD44 (86). The expression of CD44 is more frequent in circulating tumor cells than in brain metastasis. This may be contributed to the importance of CD44 in tumor cell survival in the blood flow, decline of CD44 after chemotherapy and radiotherapy (87). Sp1 is an important transcription factor in NSCLC progression, which increases in the early stage but decreases in the late stage. It can inhibit tumor stemness and metastasis. CD44 expression can be inhibited by Sp1 through inducing the expression of miR-3194-5p, miR-218-5p, miR-193-5p, miR-182-5p and miR-135-5p (88). Hyaluronan-CD44/HA-mediated motility receptor signaling pathway is overexpressed in NSCLC and associated with cell proliferation and survival (89). VCAM-1 also participates in NSCLC progression. miR-26a is downregulated in NSCLC, and it can block IL-2 mediated proliferation and migration in NSCLC by binding to the 3’-UTR binding sequence of VCAM-1 (90). Study also showed that sVCAM1 secreted by CXCR4-overexpressing NSCLC cells promoted osteoclastogenesis (91).

## Current and potential applications of cell adhesion molecules on NSCLC

### Anti-PD-1/PD-L1

PD-1/PD-L1 is an immune checkpoint and has been targeted by several monoclonal antibodies. Atezolizumab, avelumab and durvalumab are PD-L1-targeted while the other monoclonal antibodies mentioned in this paragraph are PD-1-targeted (**Table 2**). The addition of atezolizumab to bevacizumab plus chemotherapy significantly improved PFS and OS of metastatic non-squamous NSCLC patients and the Phase 3 IMpower132 study showed that combination of atezolizumab and carboplatin/cisplatin and pemetrexed significantly improved PFS (median 7.6 vs 5.2 months, HR = 0.60, 95%CI: 0.49–0.72, p < 0.0001) of non-squamous NSCLC than that of carboplatin/cisplatin and pemetrexed while statistical difference in OS had not been observed (92, 95). Atezolizumab also outcompeted docetaxel in OS for previously treated NSCLC patients (93, 96). Compared with docetaxel, avelumab failed to prolong OS in patients with platinum-treated PD-L1^+^ NSCLC. However, after 2 years follow-up, avelumab showed better OS than docetaxel especially in high PD-L1 expression subgroups (PD-L1≥50%) (99, 100). Durvalumab prolonged PFS and OS compared with placebo and this effect was further observed after 3 and 4 years, indicating the long survival benefit of durvalumab (94, 101, 102). However, durvalumab failed to statistically improve OS compared with chemotherapy in metastatic NSCLC first-line treatment (103). Cemiplimab also significantly improved OS and PFS compared with chemotherapy in patients with advanced NSCLC (PD-L1>50%) (97). Compared with docetaxel, nivolumab prolonged OS of previously treated NSCLC in predominantly Chinese patient subgroup after 2 years, which is consistent with the outcome of CheckMate 017 (104, 105). Several studies have showed that pembrolizumab plus chemotherapy improved OS compared with chemotherapy. Pembrolizumab also outcompeted chemotherapy in OS (98, 106–110). Sintilimab and tislelizumab are two Chinese original anti-PD-1 monoclonal antibodies. They are more affordable and cost-effective for Chinese patients compared with foreign drugs (111, 112). They also showed improvement of PFS in combination with chemotherapy compared with chemotherapy alone. However, OS was not obtained and more studies are needed (113, 114).

**Table 2:** Completed phase 3 NSCLC clinical trials of PD-1/PD-L1 and CTLA-4-targeted monoclonal antibodies in recent 5 years.

Agent	Intervention	Number of patients (n)	ORR (95% CI or p value), (%)	Median PFS (95% CI or p value), (months)	Median OS (95% CI or p value), (months)	Target	Reference
Atezolizumab	atezolizumab plus bevacizumab plus carboplatin plus paclitaxel bevacizumab plus carboplatin plus paclitaxel	692	63.5 (58.2–68.5) 48.0 (42.5–53.6)	8.3 (7.7–9.8) 6.8 (6.0–7.1)	19.2 (17.0–23.8) 14.7 (13.3–16.9)	PD-L1	(76) .
Atezolizumab	atezolizumab plus cisplatin or carboplatin plus pemetrexed every 21 days cisplatin or carboplatin plus pemetrexed every 21 days (induction treatment for four to six 21-day cycles) atezolizumab plus pemetrexed every 21 days pemetrexed every 21 days (maintenance treatment)	578	47 (41.1–52.8) 32 (26.8–37.9)	7.6 5.2 (p<0.0001)	18.1 13.6 (p=0.0797)	PD-L1	(77)
Atezolizumab	atezolizumab 1200 mg every 3 weeks docetaxel 75 mg/m^2^ every 3 weeks	850	14 13	2·8 (2·6–3·0) 4·0 (3·3–4·2)	13.8 (11.8–15.7) 9.6 (8.6–11.2)	PD-L1	(79)
Avelumab	avelumab 10 mg/kg every 2 weeks docetaxel 75 mg/m² every 3 weeks	529	19 (14–24) 12 (8–16)	3.4 (2.7–4.9) 4.1 (3.0–5.3)	11.4 (9.4–13.9) 10.3 (8.5–13.0) (p=0.16)	PD-L1	(81)
Durvalumab	durvalumab 10 mg/kg every 2 weeks placebo every 2 weeks	713	28.4 (24.3–32.9) 16.0 (11.3–21.6)	16.8 (13.0–18.1) 5.6 (4.6–7.8)	47.5 (38.4–52.6) 29.1 (22.1–35.1)	PD-L1	(83–85)
Durvalumab	durvalumab 20 mg/kg every 4 weeks durvalumab 20 mg/kg every 4 weeks plus tremelimumab 1 mg/kg every 4 weeks (up to 4 dose) platinum-based doublet chemotherapy	488	35.6 34.4 37.7	4.7 (3.1-6.3) 3.9 (2.8-5.0) 5.4 (4.6-5.8)	16.3 (12.2-20.8) 11.9 (9.0-17.7) 12.9 (10.5-15.0)	PD-L1	(86)
Cemiplimab	cemiplimab 350 mg every 3 weeks platinum-doublet chemotherapy	563	39(34–45) 20 (16–26)	8.2 (6.1–8.8) 5.7 (4.5–6.2)	not reached (17.9–not evaluable) 14.2 (11.2–17.5)	PD-1	(87)
Nivolumab	nivolumab 3mg/kg every 2 weeks docetaxel 75mg/m^2^ every 3 weeks	504	18 (13.6–21.9) 4 (1.7–8.5)	2.8 (2.4–3.6) 2.8 (1.8–3.0)	11.9 (10.4–13.8) 9.5 (7.6–11.2)	PD-1	(88)
Pembrolizumab	pembrolizumab 200 mg every 3 weeks saline placebo every 3 weeks	559	57.9 (51.9–63.8) 38.4 (32.7–44.4)	6.4 (6.2–8.3) 4.8 (4.3–5.7)	15.9 (13.2–not reached) 11.3 (9.5–14.8)	PD-1	(92)
Pembrolizumab	pembrolizumab 200 mg every 3 weeks saline placebo every 3 weeks	616	47.6 (42.6–52.5) 18.9 (13.8–25.0)	8.8 (7.6–9.2) 4.9 (4.7–5.5)	not reached 11.3 (8.7–15.1)	PD-1	(93)
Pembrolizumab	pembrolizumab 200 mg every 3 weeks platinum-based chemotherapy	305	44.8 (36.8–53.0) 27.8 (20.8–35.7)	10.3 (6.7–not reached) 6.0 (4.2–6.2)	not reached not reached	PD-1	(94)
Sintilimab	sintilimab plus pemetrexed and platinum placebo plus pemetrexed and platinum	397	51.9 (45.7–58.0) 29.8 (22.1–38.4)	8.9 (7.1–11.3) 5.0 (4.8–6.2)	not reached not reached	PD-1	(95)
Tislelizumab	tislelizumab plus chemotherapy chemotherapy	332	57.4 (50.6–64.0) 36.9 (28.0–46.6)	9.7 (7.7–11.5) 7.6 (5.6–8.0)	not reached not reached	PD-1	(96)
Ipilimumab	nivolumab plus ipilimumab chemotherapy	1166	35.9 (31.1–40.8) 30.0 (25.5–34.7)	7.2 (5.5-13.2) 5.5 (4.4-5.8)	17.1 (15.2–19.9) 13.9 (12.2–15.1)	CTLA-4	(97, 98)

The expression level of PD-L1 in NSCLC cells is an important criterion for PD-1/PD-L1 treatment. For pembrolizumab, PD-L1 expression≥50% is suitable for first-line therapy and ≥1% for second-line treatment, respectively. tumor is considered as PD-L1 positive if more than 50% tumor cells express PD-L1 and suitable for anti-PD-1/PD-L1 treatment. However, for the combination of chemotherapy and immunotherapy, PD-L1 expression didn’t affect survival benefit and PD-L1 expression examination is unnecessary (115). Training for pathologists may increase reliability for samples around 50% and has very little or no impact on the inter- or intra-observer reproducibility (116).

### Anti-CTLA-4

Ipilimumab is a CTLA-4-targeted monoclonal antibody which has applied in melanoma. However, it shows limited therapeutic effect and obvious adverse effect in NSCLC. Currently, all anti-CTLA-4 immunotherapy alone has limited application in NSCLC.

The effect of blocking CTLA-4 remains unclear, which limits the development of new antibodies. Traditionally, it is believed that anti-CTLA-4 antibodies produces anti-tumor effect through blocking the interaction of CTLA-4/B7. Some studies argued that anti-CTLA-4 antibodies produces anti-tumor effect by killing Tregs through antibody-dependent cell-mediated cytotoxicity because Fc domain is essential for ipilimumab anti-tumor function (117, 118). On the other hand, tremelimumab is a new CTLA-4-targeted IgG2 monoclonal antibody with less antibody-dependent cell-mediated cytotoxicity (ADCC) and also showed anti-tumor effect in clinical trials and is supported by a murine surrogate antibodies experiment, which showed that anti-tumor effect was not completely dependent on ADCC (119). Similar CTLA-4 binding properties between ipilimumab and tremelimumab also indicates the importance of CTLA-4/B7 blockage (120). Therefore, anti-CTLA-4 antibodies may produce anti-tumor effect through both CTLA-4/B7 blockage and ADCC, and other unknown mechanisms may also take effect.

### Anti-MUC1

Tecemotide (L-BLP25) is a MUC1 antigen-specific tumor vaccine to induce T cell immunity against MUC1. A phase 3 clinical trial (NCT00409188) showed that no statistical difference was observed between tecemotide group (median 25.8 months) and placebo group (median 22.4 months) after chemoradiotherapy in overall survival (HR 0.89, 95% CI 0.77–1.03, P = 0.111) for patients with unresectable stage III NSCLC. However, improvement median survival in tecemotide group was observed in the analysis of concurrent chemoradiotherapy subgroup (29.4 months versus 20.8 months, aHR 0.81, 95% CI 0.68–0.98, P = 0.026). Elevated sMUC1 and ANA might be associated with survival benefit with tecemotide (interaction P = 0.0085 and 0.0022) and might be potential biomarkers (121, 122). TG4010 is a modified vaccinia Ankara encoding MUC1 and human IL-2. Studies showed that clinical benefits of TG4010 was associated with anti-MUC1 T cell responses. And anti-MUC1 responses contributed to epitope spreading against other tumor associated antigens (123). Although there was no statistical difference in health-related quality of life between TG4010 plus chemotherapy group and chemotherapy group in (NCT00415818), TIME study (NCT01383148) showed that TG4010 and chemotherapy group (5.9 months, 95% CI 5.4–6.7) had a prolonged PFS compared with placebo and chemotherapy group (5.1 months, 95% CI 4.2–5.9) (HR=0.74 95% CI 0.55–0.98; one-sided p=0.019) (124, 125). Gatipotuzumab is an anti-tumor-associated epitope of mucin-1 monoclonal antibody and tomuzotuximab is an anti-EGFR antibody. The combination of these two drugs has been tested in the GATTO study (126). A MUC1-targeted dendritic-cell-based vaccine exhibited anti-tumor activity and clinical benefits for patients with MUC1-positive refractory NSCLC. The median survival time and 1-year survival rate for patients who received more than six vaccinations was 9.5 months and 39.3%, while those of patients received initial vaccination was 7.4 months and 25.0%, respectively. Immune-related adverse events occurrence (12.6 months versus 6.7 months, p=0.042) and peripheral lymphocytes count/white blood cells count > 20.0% (12.6 months versus 4.5 months, p=0.014) were associated with longer survival time and might be predictive biomarkers for better clinical benefits (127).

## Other molecules

High levels of CD31+ circulating tumor endothelial cells are associated with poor prognosis in anti-angiogenic therapy and may be a predictive factor (128, 129). Downregulation of CD44 is related to inhibition of wild−type EGFR signals, which leads to of cell proliferation inhibition and increased cisplatin sensitivity (130, 131). CD44 overexpression is also associated with sensitivity to PD-1 axis blockade and may be a novel auxiliary biomarker (132). These findings indicate that CD44 blockade may facilitate anti-PD-1 therapy and cisplatin treatment efficacy. The levels of serum VCAM-1 are significantly higher in NSCLC patients, which be a potential auxiliary biomarker (133). High baseline serum levels of VCAM-1 are related to better prognosis in NSCLC patients treated by second line nivolumab and may also be a potential biomarker for anti-PD-1 therapy (134).

## Combined therapy

For advanced NSCLC, one therapy alone may be not enough to reach satisfactory therapeutic effect. Therefore, the combination of immune checkpoint blockers and other therapies may improve treatment efficacy and has been carried out by multiple studies (135, 136). The combination of radiotherapy and anti-CTLA-4 therapy activates T cells with TCR against tumor cells repertoires and the combination of immune checkpoint inhibitor and radiation/chemotherapy has been clinically applied in unresectable stage III NSCLC (18, 137). Studies showed that the combination of nivolumab and ipilimumab (dual checkpoint inhibition) significantly improved OS and PFS in NSCLC and especially effective for high mutational burden tumors and PD-L1 expression <1% (138–141). Although nivolumab plus ipilimumab seemed to outcompete other ICI therapies in OS for patients with PD-L1 expression <1%, the risk of Grade 3 or higher treatment-related adverse effects was also significantly increased compared with nivolumab alone (OR=5.80; 95%CI, 1.60-21.0). Therefore, the clinical benefit and tolerance of patient should be evaluated before dual checkpoint inhibition administration and more studies are needed (142). A case report showed that cryotherapy is a feasible treatment for ICI resistant metastasis (143). The combination of nivolumab and ipilimumab (dual immune blockage) may be more efficient than nivolumab or ipilimumab alone (144). The combination of low-dose apatinib (VEGFR2-TKI) and anti–PD-1 also improved treatment efficacy in NSCLC mouse model and patients (145). Evodiamine inhibits the MUC1-C/PD-L1 axis and enhances CD8+ T cells function, which may be combined with ICIs in NSCLC to enhance treatment efficacy (146). A network meta-analysis of 7155 NSCLC patients showed that the combination of chemotherapy plus PD-L1 inhibitors plus dexamethasone pretreatment may be a candidate for the first-line treatment of NSCLC patients (147). Combined therapy is also a potential method to overcome ICI resistance. The combination of oncolytic viruses and ICIs showed to overcome ICI resistance in a syngeneic mouse model and a case report (148, 149). T-cell immunoglobulin domain and mucin domain-3 (TIM-3) is an inhibitory receptor associated with PD-1 and may also participate in anti-PD-1 drug resistance. A study showed that high TIM-3 level on tumor infiltrating lymphocytes was related to poor prognosis in NSCLC (150). Therefore, the dual blockade of PD-1 and TIM-3 may overcome anti-PD-1 drug resistance. A clinical trial of the combination of sabatolimab (anti-TIM-3 antibody) and spartalizumab (anti-PD-1 antibody) has also been carried out and showed some signs of anti-tumor immunity (151).

Chimeric antigen receptor-T cell (CAR-T) expresses a modified protein containing three regions: extracellular region which can specifically bind to tumor cells, transmembrane region and intracellular region which can induce stimulatory signal transduction. This unique receptor leads to the robust anti-tumor effect (152). The combination of checkpoint inhibitors and CAR-T cell therapy may further activate anti-tumor immunity and are being applied in many clinical trials (153). However, CAR-T cell therapy has limited efficacy for most solid tumors. For NSCLC, it has following shortcomings: toxicity induced by CAR-T cell; lack of adequate tumor-specific antigens as targets and poor anti-tumor effect (154). EGFR and VEGFR2 may be potential target antigens for CAR-T cell therapy against NSCLC (155). CAR-T cells with constitutively anti-PD-1 secretion also showed better anti-tumor activity in mouse model (20).

The objective of tumor vaccine is to promote an activate adaptive anti-tumor immune response (156). However, outcomes of NSCLC vaccine clinical trials were disappointing. NSCLC vaccines should overcome the inhibition of tumor microenvironment, negative checkpoint signals and the low availability of effector T cells to tumor sites (157). Neoantigens is a class of tumor-specific antigens caused by non-synonymous mutations of tumor cells. Unlike tumor-associated antigen, it is not expressed in normal cells and induces stronger immune reactions, which may be a promising peptide target for individualized tumor vaccine (158, 159). The combination of ICIs and tumor vaccine may enhance treatment efficacy and has been used in clinical trials (156).

Not all combined therapies are adequate. The combination of immunotherapy (except immune checkpoint inhibitors) and conventional treatment didn’t improve prognosis and might increase incidence of adverse events (15). Several clinical trials also failed to show a clinical benefit of PD-1/PD-L1 monoclonal antibodies in patients with EGFR-mutant NSCLC (160). These studies indicate that combined therapy should be used carefully in order to reach a balance between clinical benefit and adverse events.

## Adverse effects and drug resistance in immunotherapy

### Adverse effects

Adverse effects also happen in anti-PD-1/PD-L1 immunotherapy. Interestingly, according to a small sample study, the incidence rate of irAEs was significantly higher in responders than non-responders (65.2% vs. 19.3%, P < 0.01), indicating that irAEs may be related to better therapeutic effect (161). Whether anti-PD-1 or anti-PD-L1 antibodies has less adverse events remains controversial and studies showed contradictory outcomes (17, 162–164) A study showed that HLADRB1*11:01 and pruritus as well as a nominally significant additive association between HLA-DQB1*03:01 and colitis, indicating the role of genetic diversity in adverse events of immune checkpoint inhibitors (165). Cardiovascular toxicity is a rare but serious adverse event of immune checkpoint inhibitors treatment, including myocarditis, pericarditis, heart failure, acute myocardial infarction, etc (166). Pneumonitis is also a common adverse effect in anti-PD-1/anti-PD-L1 treatment for NSCLC patients with high mortality and poor treatment (94, 167, 168). Interstitial lung disease is a rare (1–5%) but severe adverse effect (50–60% mortality rate). A study indicated that performance status≥ 2 and ≥ 50 pack-year were independent risk factors of ICI-induced interstitial lung disease of grade≥ 3 and all grades (169). Different therapy showed different distribution of irAEs. Atezolizumab plus platinum may be related to a higher incidence rate of colitis, while pembrolizumab may be related to a higher incidence of pneumonitis and hepatitis (170). Irrational drug use also increases the frequency of adverse effects. A study showed that the use of osimertinib immediately after nivolumab is associated with higher incidence of grade 3 or higher hepatotoxicity in patients with advanced NSCLC harboring EGFR mutation acquired T790M resistance (171). Sintilimab monotherapy showed a higher incidence of fatal adverse effects (0 to 2.5%) than average (172). Lower dose and frequency of ipilimumab seems to improve the safety of combined therapy (139). Hyperprogression is defined as an unexpected acceleration of cancer growth after immunotherapy and is a severe adverse effect with bad prognosis. NSCLC has a relatively high incidence rate of hyperprogression (14%) and no biomarker has been identified (173). High level of IL-10 and low pretreatment neutrophil-to-lymphocyte ratio may be predictive factors for irAEs monitor (174, 175).

### Drug resistance

**Figure 2:**
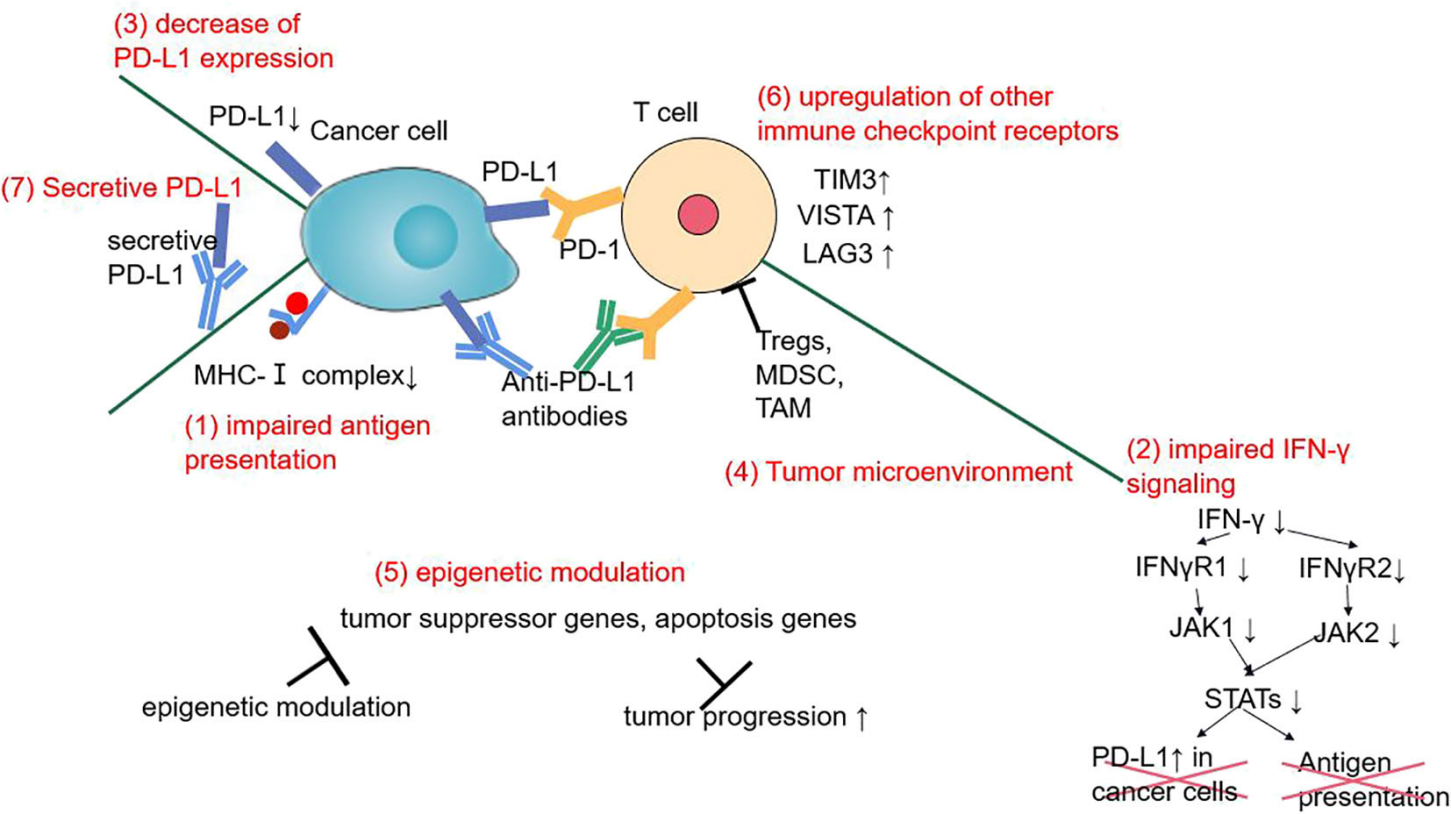
The mechanisms of primary or secondary ICI resistance in NSCLC during anti-PD-1/PD-L1 therapy.

Primary or secondary drug resistance has been observed in NSCLC anti-PD-1/PD-L1 therapy. ORRs are no more than 20–30% and no biomarker (except PD-L1) has been identified. Underlying mechanism may include tumor neoantigen loss, impaired IFN-γ signaling, upregulation of other immune checkpoint receptors, tumor microenvironment, epigenetic modulation, etc (176). Secretive PD-L1 may also be one of the resistance mechanisms by combining with anti-PD-L1 antibody (177). An autopsy of two immunotherapy failure patients also showed a sharply decrease of PD-L1 expression after immunotherapy (178). Acquired EGFR exon 21 L858R has been observed in a nivolumab resistant patient, which may also be a possible mechanism of secondary ICI resistance (179) (**Figure 2**).

There are some characteristics in anti-PD-1/PD-L1 resistance and may be future biomarkers to predict treatment efficacy. Tumor Mutation Burden (TMB) and EGFR/ALK mutations are two promising biomarkers (180, 181). Several studies have showed that patients with high tissue TMB have a higher ORR with immune checkpoint inhibitors than those with low tissue TMB (182, 183). EGFR-mutated NSCLC, on the other hand, is related to lower PD-L1 expression, low TMB, and increased risk of hyperprogression and pulmonary toxicity (184). ALK-/EGFR-positive tumors are negative prognostic factors in immunotherapy and immunosuppressive microenvironment has also been observed in ALK/EGFR-positive tumors (185). Whether KRAS is a good prognostic factor in ICI therapy remains controversial (186). Low blood serum amyloid A may also indicate better survival outcomes for pembrolizumab in advanced NSCLC treatment (187). High levels of serum hypoxanthine and histidine may be associated with better anti-PD-1 response (188). Activation of Hedgehog pathway, increasing of plasma Wnt1 protein, TGFBR2 mutation and CDKN2A loss−of−function are also associated with ICIs resistance in NSCLC (189–191). A study showed that high level of circulating monocytic myeloid-derived suppressive-like cells was related to primary resistance to immunotherapy (192). Interestingly, smoking patients with NSCLC seemed to have better responses to anti-PD-1/PD-L1, anti-CTLA-4, and anti-MUC1 drugs compared with non-smokers, which might affect therapy selection for smokers with NSCLC (193).

## Prospect

Aberrant cell adhesion molecule expression plays a crucial role in tumor microenvironment formation and tumor immune evasion which can be utilized in immunotherapy. Upregulation of PD-1/PD-L1/CTLA-4 leads to tumor immunosuppression in NSCLC. Downregulation of E-cadherin facilitates tumor metastasis and LFA-1 plays a complex role in tumor progression. MUC1 upregulation affects various signaling pathways to enhance the proliferation, migration and invasion ability of NSCLC. Currently, ICIs has made great progress while other immunotherapies showed disappointing efficacy in NSCLC. Primary or secondary drug resistance and irAEs are still major barriers for ICIs.

For immune checkpoint inhibitors, the expression level of target molecules is important for drug selection. Novel laboratory technologies such as On‐chip Sort (a microfluidic chip cell sorter) can separate and analyze circulating cancer cells, which is convenient, non-invasive and more sensitive compared with biopsy (194). The delivery strategy of anti-tumor drugs is associated with treatment efficacy and adverse effects, and cell adhesion molecules may be a promising target (195). αVβ3 integrin overexpression in tumor is a sign of angiogenesis. Therefore, RGD peptides may be peptide-drug conjugate because it can selectively bind to cancer cells with αVβ3 and αVβ5 integrins highly expressed (196). The combination of immunotherapy and other treatments may improve therapeutic effect. However, combined therapy may also lead to increased adverse effect and should be used carefully on the basis of evidence-based medicine. Dual immune checkpoint inhibitors (the combination of anti-PD-1 and anti-CTLA-4 therapy) is considered as a promising therapy to enhance ICI efficacy and showed improved prognosis in melanoma and NSCLC (139, 197). CAR-T cell immunotherapy is mainly used in hematological tumors and not being clinically applied in NSCLC. Future CAR-T cell immunotherapy for NSCLC should be more specific in tumor antigens selection and TCR designation in order to robustly inducing anti-tumor effect and decreasing the incidence adverse effects (20, 198, 199). ICAM-1 specific CAR-T cell may also increase its efficacy and safety (200). The combination of ICI (exogenous or secretive) and CAR-T cell therapy may further activate anti-tumor immunity (20, 153).

Despite of great progression, NSCLC is still a deadly and highly heterogenous disease. One or two therapies can hardly control disease progression and drug resistance is easily developed. Therefore, individualized and combined therapy are further trends for NSCLC immunotherapy. To overcome the drawbacks in immunotherapy, understanding the mechanisms of cell adhesion molecule interaction; new biomarkers for diagnosis, monitor and follow-up; novel technologies and therapies; and accumulation of clinical experience are necessary for a wider application of NSCLC immunotherapy.

## Author contributions

HY, YM designed and conceptualized the review. ZY, MW and XJ provided precious advice and corrections. All authors contributed to the article and approved the submitted version.
